# Realities, perceptions, and strategies for implementation of an ethical population management program for dogs and cats on university campuses

**DOI:** 10.3389/fvets.2024.1408795

**Published:** 2024-07-24

**Authors:** Gustavo Canesso Bicalho, Lucas Belchior Souza de Oliveira, Camila Stefanie Fonseca de Oliveira, Adriane Pimenta Da Costa Val Bicalho, Camila Valgas Bastos, Camila Machado Torres, Christina Malm, Fernanda Louro de Souza, Graciela Kunrath Lima, Lorena Diniz Macedo Silva Maia, Luiz Carlos Villalta, Marcelo Pires Nogueira de Carvalho, Rossimiriam Pereira de Freitas, Vania Regina Goveia, Werik dos Santos Barrado, Yara de Freitas Oliveira, Danielle Ferreira de Magalhães Soares

**Affiliations:** ^1^Veterinary Epidemiology Laboratory, Preventive Veterinary Department, Veterinary School, Federal University of Minas Gerais (UFMG), Belo Horizonte, Minas Gerais, Brazil; ^2^Department of Veterinary Clinic and Surgery, Veterinary School, Federal University of Minas Gerais (UFMG), Belo Horizonte, Minas Gerais, Brazil; ^3^Environmental Management Department, Federal University of Minas Gerais (UFMG), Belo Horizonte, Minas Gerais, Brazil; ^4^Environmental and Biosafety Management Department, Veterinary School, Federal University of Minas Gerais (UFMG), Belo Horizonte, Minas Gerais, Brazil; ^5^History Department, College of Philosophy and Human Sciences, Federal University of Minas Gerais (UFMG), Belo Horizonte, Minas Gerais, Brazil; ^6^Chemistry Department, Institute of Exact Sciences, Federal University of Minas Gerais (UFMG), Belo Horizonte, Minas Gerais, Brazil; ^7^Basic Nursing Department, Nursing School, Federal University of Minas Gerais (UFMG), Belo Horizonte, Minas Gerais, Brazil

**Keywords:** veterinary public health, one health, community animals, perception evaluation, campus university

## Abstract

Stray dogs and cats pose significant challenges for public health and animal welfare due to their potential involvement in zoonotic disease transmission, accidents, and aggressions. Large urban centers exacerbated challenges due to the presence of these animals in public areas with high human density. Ethical Population Management Programs (EPMP), rooted in the One Health approach, are crucial for addressing this issue comprehensively. This study aimed to demonstrate the approach on cats and dogs EPMP and evaluate the perceptions of academic community regarding EPMP implementation on a campus situated in urban territory. The study was conducted at the Pampulha campus of UFMG in Belo Horizonte, Brazil. In response to issues of animal abandonment and conflicts, the Permanent Commission for Animal Policies (CPPA-UFMG) was established in 2019 to manage the campus’s dog, cat, and wildlife populations. The commission implemented the Trap-Neuter-Return (TNR) method, along with health assessments and vaccinations for animals. Interviews were conducted with campus staff to gauge their perception of animal management strategies. Retrospective and prospective analyses of the commission’s actions were carried out to assess implementation processes and challenges. The animal population survey conducted on campus between July 2018 and September 2021 revealed a total of 266 animals recorded. Among these animals, 195 were cats (73.3%) and 71 were dogs (26.7%), with the majority being adults. Subsequent surveys in 2019 and 2021 showed a slight increase in the animal population, with measures such as sterilization contributing to population control. Perception analysis among campus users indicated strategies such as TNR were widely endorsed for population control. The employees perception questionnaire was applied to 115 individuals, representing 42 units/departments and five gates. Associations were found between these beliefs and support for institutional actions. The majority favored sterilization (92.17%) and agreed that TNR is an appropriate approach to population control. Overall, the study reflects a community concerned about animal welfare and supportive of measures to address population management and cruelty prevention. The continuous efforts of the university’s CPPA have led to stability in the resident animal population, indicating success in achieving population control objectives.

## 1 Introduction

Stray dogs and cats lacking basic health care assistance represent a challenge for public health management and animal welfare, as those animals could be involved in zoonotic disease transmission, accidents, aggressions such as bites and scratches, as well as property damage (1–4). It is estimated that 10% of dogs in urban and rural areas in Brazil do not possess a responsible owner, with significant variations, potentially reaching values close to 37% (5).

The challenge is exacerbated in large urban centers, where these animals are present in public areas with high human density (6). University campuses, for example, when they have large territorial extensions and many preserved green areas, may facilitate abandonment, and even though they are not suitable places for domestic animals to stay, many of them, after being abandoned, survive, and may be involved in academic community conflicts. As a solution, Ethical Population Management Programs (EPMP) are necessary, which should be based on the premise of One Health, that is, the interface between human, animal, plant, and ecosystem health (7), and therefore encompass actions that consider political, behavioral, ecological, sanitary, and socio-environmental aspects (3).

A globally disseminated method of ethical population management, especially for the control and stabilization of feral cat colonies, is known as Trap-Neuter-Return (TNR). This technique consists of a non-lethal strategy, where the animal is captured, surgically sterilized, identified, registered, and returned to its original community. The method exists because it is not feasible to promote the adoption of all stray animals and relies on the Vacuum Effect Theory, which states that by permanently removing animals from an environment, if there are other stray animals, there would be an influx of new individuals with unknown reproductive and health conditions, leading to an uncontrolled cycle, as the location allows these new animals to benefit from the environmental conditions necessary for life maintenance, such as shelter, water, and food (8, 9). There are variations of TNR that involve vaccination, deworming, and other necessary veterinary care, such as testing for Feline Immunodeficiency Virus (Fiv) and Feline Leukemia Virus (FeLV), rabies vaccination, sterilization, and returning to the original location for cats (10, 11).

TNR has been the method of choice for managing the population of cats on university campuses in other countries, such as in the USA at Texas A&M University (12) and the University of Central Florida (13); at the University of KwaZulu-Natal in South Africa (14); at the American University of Beirut and Lebanese American University in Lebanon (11); and at the University of South Wales in Australia (10).

Although less common, there are studies in the literature that demonstrate TNR programs involving dogs in urban centers, such as in the Hong Kong SAR (Special Administrative Region), where the Society for the Prevention of Cruelty to Animals (SPCA) managed to sterilize 75% of the free-roaming dog population and return all that were possible to their original location (15); and in Greater Bangkok, which in 5 years of high-intensity catch, neuter, vaccinate and return interventions, the free-roaming dog density was reduced by 24.7%, while the monthly average of canine rabies cases reduced to 5.7% (16).

Despite the various possibilities associated with campus domestic animal management practices, the methods applied should be evaluated from perspectives of qualitative and quantitative indicators, which are included as important measures in veterinary epidemiology (17). According to Garcia, Calderón, and Ferreira (3), diagnosing the situation is the first step in building a population management program, as it allows for understanding the reality on which one intends to act and proposing interventions. For this, it should involve data collection on the animal population (population dynamics) and human attitudes and behaviors regarding animals.

Qualitative and quantitative methods of health indicators are important, especially in evaluating changes after the implementation of certain practices, signaling whether the proposed objectives were or are being conducted appropriately (18, 19). In Brazil, the humane and ethical management of animals in institutionalized settings is not a reality in most universities. The Federal University of Minas Gerais, located in a Brazilian metropolis, pioneered the creation of its Policy for Ethical Population Management of Stray Domestic Animals and Wildlife Surveillance on Campuses in 2018, opting for the TNR method along with other actions, thereby establishing a comprehensive yet effective Program for Humane and Ethical Management of Domestic Animals.

This study aimed to demonstrate the approach on cats and dogs population management and evaluate the perceptions of university security guards, cleaning professionals, and animal caretakers regarding EPMP implementation on a campus situated in urban territory.

## 2 Materials and methods

### 2.1 Study area

**Figure 1:**
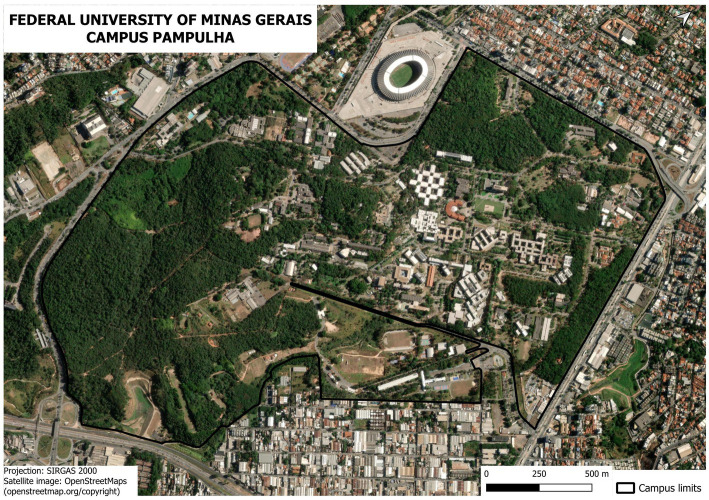
UFMG’s Pampulha campus map with units and entrances which the questionnaire was applied.

The study was conducted on the Pampulha campus of the UFMG (Figure 1), founded in 1962, which houses one of the largest green areas in the city of Belo Horizonte, Minas Gerais, Brazil. Its terrain covers approximately 3.3 km^2^, accommodating 22 academic units, 21 administrative units and their departments, as well as communal and utility spaces such as university restaurants and the service plaza. Around 50,000 people circulate through the Pampulha campus on a typical school day (20). It is surrounded by urban, residential, and commercial areas inhabited by people from various levels of social vulnerability.

There are reports that sighting of new animals on the campus has always occurred, as well as conflicts arising from their presence, sometimes resulting in serious cases of mistreatment of dogs and cats, as well as attempts at mass extermination, even in relatively recent times.

In mid-2018, in response to the scenario of abandonment and conflicts with the presence of animals, a commission was created by the university administration to draft a policy for the university, and in 2019 this became the Permanent Commission for Animal Policies of UFMG (Comissão Permanente de Políticas de Animais, CPPA-UFMG), aiming for an ethical population management of dogs and cats residing on the campus, as well as the wildlife population vigilance (21). In 2020, the actions of the Commission were formalized through an Extension Program composed of collaborators, including faculty, students, and administrative staff. For management, the decision was made to implement the TNR method, including testing cats for Feline Immunodeficiency Virus (FIV) and Feline Leukemia Virus (FeLV), and dogs for visceral leishmaniasis (VL), all through immunochromatography-based screening tests, resorting to confirmatory serological testing if necessary, and rabies and species-specific polyvalent vaccination, ear-tipping of cats, placement of identification collars on dogs, and subcutaneous implantation of microchips in both species. Thirty-four feeding points were defined and standardized on the campus, in less visible locations, with daily maintenance by volunteer members of the commission.

Among the main objectives of the Management Program are increasing life expectancy, a common goal of community animal programs; increasing community engagement; reducing births, diseases, and deaths; and decreasing abandonment.

### 2.2 Animal population survey

Once a year, a census was conducted across the entire campus area using three methodologies:

- Notification of each animal by appointed collaborators (unit’s directors, gatekeepers, and university security guards) through a messaging app to register all animals present in the unit during the month of September yearly.
- On-site observation for 1 week, at two different moments (morning and evening), 5 days a week, with two researchers walking and driving along the university route, taking photographic records of the animals sighted.
- Installation of cameras trap at feeding sites in each unit to record elusive animals.

The created database contained information about the animal (species, apparent age, sex, apparent health status, behavior, reproductive condition), date, time, and the unit or area where it was sighted. Subsequently, as other actions were taken regarding animal’s management, new data were recorded, such as the date of surgical sterilization, vaccination, deworming, and outcomes (release, adoption, referral to temporary home, euthanasia, and death).

### 2.3 Perception of users and employees

Interviews were conducted with university security guards, cleaning professionals, and gatekeepers, with a sample calculation to define a simple proportion using the Epitools tool^1^ according to Thrusfield (22). The calculated sample size was a minimum of 97 respondents, considering an infinite population size and a 10% margin of error. This minimum number was proportionally divided among the campus staff (Table 1).

**Table 1:** Minimum number of questionnaires to be administered, according to sample calculation, to employees in the security, gatekeeping, and cleaning sectors of the Pampulha campus of UFMG, 2021.

Employees on Pampulha campus, UFMG			
Position	Campus sample	%	Minimum sample to be applied
Cleaning professionals	231	31.77%	31
Security guards	156	21.46%	21
Gatekeepers	340	46.77%	45
Total	727	100.00%	97

Regarding the existing commission members until the year 2021 (*N* = 18), according to the sample calculation, at least two interviewees would be necessary. However, due to the ease of access and availability of individuals to participate in the study, all representatives from campus units were interviewed. The study period coincided with the coronavirus pandemic (SARS-CoV-2) and coincided with the presence of only essential activities on the Pampulha campus, justifying the selection of these professionals for the perception research as they were present in their units during the first year of Program implementation.

A semi-structured questionnaire containing 39 questions was developed (Supplementary material). A pilot test was conducted with collaborators from the School of Veterinary Medicine at the UFMG to ensure it could be administered by interviewers and understood and answered by respondents (23).

Variables related to the location (unit or work area of the interviewee and whether it was associated with the Commission), demographic aspects such as position/function, gender, age group, education level, and length of service in the location were analyzed, as well as perception regarding population management methodologies of dogs and cats and responsible pet ownership.

The interviews took place between September 8th and October 5th, 2021, in person.

### 2.4 Implementation of the commission analysis

A retrospective analysis was conducted on the commission’s actions from its inception in July 2018 to September 2021, using documents generated from meetings and activities, as well as gathering information through dialogues and discussions with long-standing commission members. Additionally, a prospective monitoring was carried out from October 2021 to December 2023, tracking the actions and meetings of the CPPA. All produced content was recorded in a database and utilized to generate information about the commission’s implementation process, including observed challenges, strengths, and the consolidation of actions thus far.

### 2.5 Data analysis

The data was tabulated and subjected to frequency analysis, followed by Pearson’s chi-square and Fisher’s exact tests to verify associations. If significant, pairwise comparisons were conducted using standardized residual analysis. Associations were considered significant when the residuals exceeded 1.96 (24). All analyses were performed using Stata/MP version 16.0 (STATACORP LLC), and significance was set at *p* ≤ 0.05.

## 3 Results

### 3.1 Implementation of the dog and cat management program at UFMG

The records of documents and meetings from the Commission demonstrated that, by 2023:

1. Meetings were held with all directorates to sensitize unit managers and appoint collaborators in each of them.
2. Subcommission were created so that each collaborator could perform specific functions within the Commission (Animal registration and identification, Feeding, Animal population management, Clinical and surgical management, Animal disposition/placement, Wildlife monitoring, Education, and Financial).
3. The appointment of collaborators was done according to everyone’s involvement and skills in animal welfare.
4. Standardization and monitoring of feeding points by caregivers in each unit were implemented.
5. Continuous education initiatives on health and animal welfare were promoted for the collaborators.
6. Actions to deter abandonment were carried out: permanent signs at the five main campus’ entrances, gatekeepers training, and, educational initiatives focusing on the academic community, including the distribution of an online handbook.
7. Ensure a quick pickup, sterilization, identification, and release of newly sighted animals: sterilization was covered by University, using funds allocated by the Rectory, while some animals were also included in the Sterilization Program run by the local City Hall or through partnerships with veterinary clinics.

Continuously, educational actions for the academic community and the external public were carried out, in partnership with the municipal public service, as the origin of most of the animals was abandonment, and they found access to food, water, and shelter on campus, with conditions for survival, even if not always ideal. Furthermore, signs were implemented at all entrances, informing about the criminal nature of animal abandonment.

Among the facilitating points identified in the implementation of the UFMG Management Program, the following stood out:

- Establishment of a Permanent Commission by the university’s board, which ensured the continuity of actions regardless of the occupants of the position and the philosophy of each administration;
- Appointment of collaborators in each academic or administrative unit, all designated by the management and with acceptance mediated by signing a Free and Informed Consent Form, in order to institutionalize the process of surveillance and care for the animals;
- Formalization of an Extension Program in the University’s Extension Board Office, in order to ensure certification for participation time to all commission collaborators;
- Division of tasks into eight working subcommission (registration and identification, feeding, population management, clinical and surgical management, destination, wildlife vigilance, education, and finances), allowing collaborators to organize themselves into groups according to their skills and preferences;
- Collaboration of the university veterinary hospital on the Pampulha Campus, which plays a significant role in the actions of the Management Program;
- Partnership with the Belo Horizonte City Hall, through the Center for Zoonosis Control;
- Cooperation from the Integrated Residency Program in Veterinary Medicine on the UFMG Pampulha campus, with active participation of Public Health residents in all actions of the Permanent Commission.

Among the challenging points highlighted during the implementation of the UFMG Management Program:

- Difficulty in managing personal relationships and divergent thoughts within the group of 45 volunteers, who had different professional backgrounds and distinct beliefs regarding animal care. The solution to minimize this issue was to conduct training sessions to level up everyone’s knowledge, especially on the T.N.R. technique, the no-shelter policy, and ear tipping, which sparked considerable prejudice due to a lack of technical understanding;
- Motivating the 45 volunteers to actively participate in the Program, as some were appointed by the directorates but were not inherently motivated. While they were a minority, their lack of engagement triggered comparisons among more active members, leading to feelings of overload and fatigue risk. The solution to this problem was to hold private meetings with some volunteers and the directorates, urging them to take responsibility for the animals in their unit;
- Resource acquisition: undoubtedly, this is the major obstacle to the Management Program’s actions. After receiving the initial funding in 2020, which lasted a year and was used to manage 100 animals, there was a need to supplement the resources with new strategies devised by the commission. The annual management plan sent to the University board did not include provisions for clinical and surgical care for campus animal incidents, such as vehicular trauma, severe illnesses, and other affections. Therefore, sponsorships and new fundraising methods were necessary. The strategy to alleviate the resource shortage was to establish a financial subcommission to plan and manage resources from promotional events and new sponsorships.
- Controlling new abandonments: due to the campus’s vastness, pandemic isolation, and lack of surveillance cameras at various points, new abandonments were still observed at UFMG. The solution to this problem was intensive training for surveillance, gatekeeping, and cleaning professionals, along with the installation of anti-abandonment signs and improvements to surveillance cameras in each unit.

### 3.2 Animal population survey

**Figure 2:**
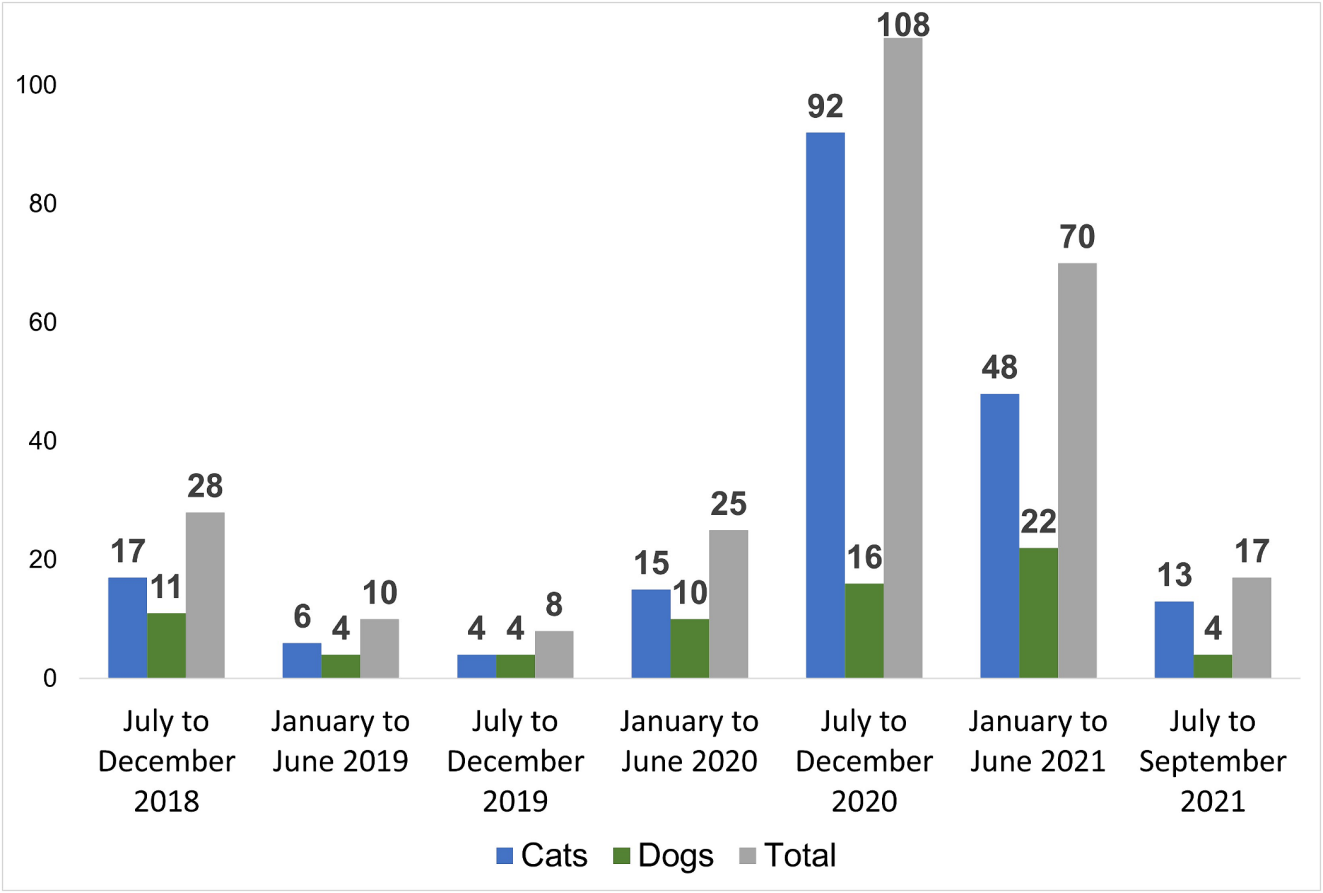
Number of new entries into the database by species between July 2018 and September 2021.

Among the quantitative data from the work carried out on campus, from July 2018 to September 2021, 266 animals were recorded (approximately 6.8 new animal records per month), in 25 different units or areas, of which 195 were cats (73.3%) and 71 were dogs (26.7%), with 71% (189) adults. All the animals were new sightings, as the method of photographic recognition with confirmation by the caretakers and commission members was used, resulting in the insertion of new entries into the database based on this criteria. Figure 2 shows the number of new sightings of dogs and cats recorded in our database by period, and it is important to note that animals sighted previously or that have settled on the campus are not included in subsequent periods. There is no information about the destination of all the animals sighted on campus during the study period, since many were seen only once, which may indicate that they passed through the university space, were recorded in the database, but did not settle there.

Surgical sterilization, rabies and polyvalent vaccination were performed, as well as FIV/FeLV tests in felines and visceral leishmaniasis in canines, for 97 cats (38 males and 59 females) and 27 dogs (13 males and 14 females). Through the CPPA, 47 adoptions (17.7%) were mediated during the period, with 38 cats and 9 dogs. There was a 26% increase in adoptions in the first year of the CPPA-UFMG, compared to the year before its implementation.

The units have established locations for feeders and drinkers for the animals, which are standardized and monitored by the Commission, as well as the establishment of protocols and flowcharts, including feeding practices and water supply, and addressing events related to animal health. Educational activities for the academic community and the public have been developed in partnership with the municipal public service, as the origin of most animals was abandonment or migration from nearby regions, and they found access to food, water, and shelter on campus, conditions for survival, even if not always ideal. Conflict management among professors, animal protection technicians, and campus employees was resolved through access to information, dialogue, and standard operating procedures. There was an urgent need for the implementation of actions to prevent animal abandonment on campus. Signs were implemented at all entrances, and educational activities are ongoing consistently.

The first survey conducted in 2019 estimated that there were approximately 100 animals on campus, with 80 cats and 20 dogs. In the census conducted in 2021, through consultation with local representatives and unit caregivers, it was found that there were 104 animals in 21 campus units (Supplementary material), including 91 cats (41 males, 38 females, and 12 with no sex information) and 13 dogs (6 males and 7 females). There was an increase of four animals in 2021 compared to the census conducted in 2019. Considering the more efficient surveillance since the implementation of the Program, which increased the number of sightings, the small increase in the number of animals suggests a trend towards population stability on campus, probably with a significant influence from the activities carried out by CPPA-UFMG.

The sex ratio (male:female) among cats was 1.26:1 and among dogs was 0.86:1. Among the cats, 92.3% (84) were adults, and all dogs were adults. Regarding the number of animals present on campus in the 2021 census, 65.38% (68) were surgically sterilized. Of the total cats, 60.4% (55) were sterilized (80.5% of males and 57.9% of females). All dogs were surgically sterilized.

**Figure 3:**
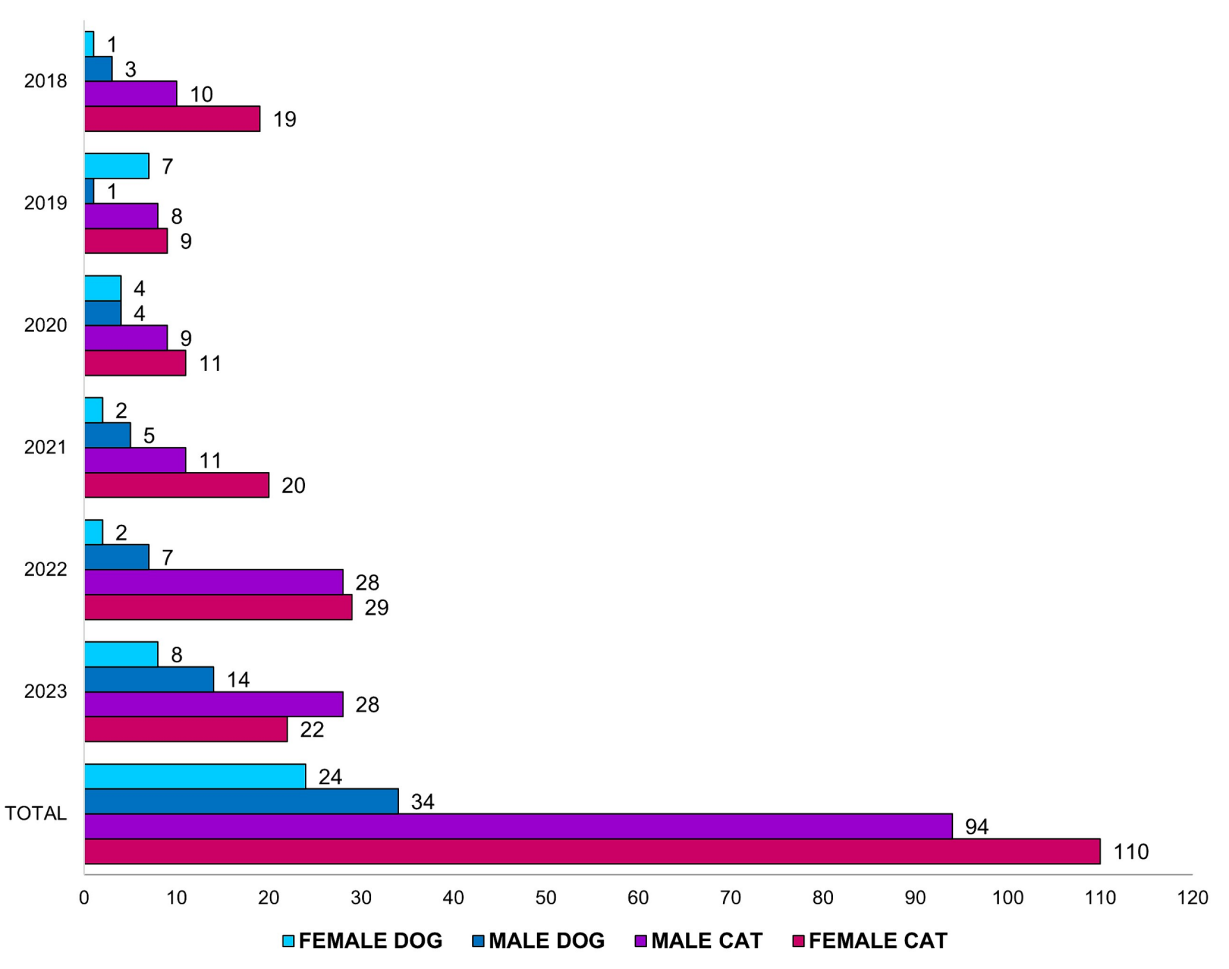
Quantitative of sterilizations per year, by species and sex.

In the prospective study, in 2022, a new census was conducted on campus, covering all units, revealing 125 resident animals at 34 sighting points (117 cats and 8 dogs). A 100% sterilization rate was observed for these animals, as some had already been neutered in other periods at the time of the census, and all were vaccinated against rabies. Figure 3 demonstrates the number of animals neutered by the committee per year. This high sterilization rate was made possible through visits to the different focal points of the colony, dialogue with caretakers, monitoring by trap cameras to better understand the dynamics of movement within the territory, use of automatic activation Brazilian traps for capture, use of the manual drop trap DT1 from the company Tomahawk Live Trap for animals that did not enter the automatic trap, and a routine of night-time captures.

Five years after the implementation of the Commission, another census was conducted in 2023, and stability of the resident population on campus was observed – *N* = 95 animals (83 cats and 12 dogs), indicating that the continuous and systematic actions adopted by the CPPA are successfully achieving the population control objective.

### 3.3 Perception analysis regarding population ethical management actions of dogs and cats

**Figure 4:**
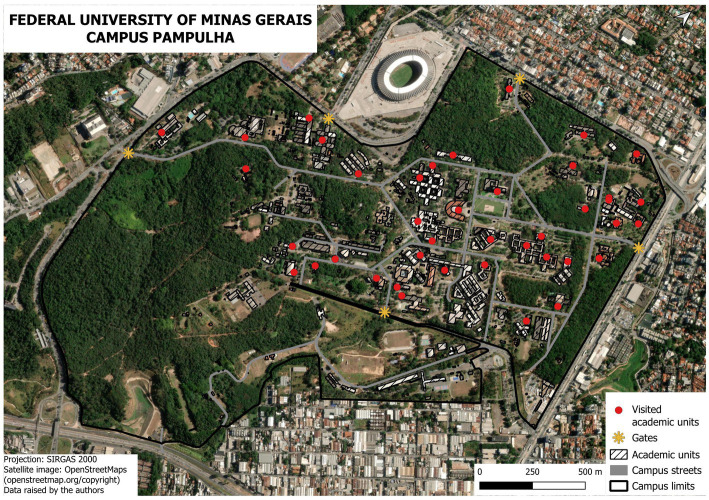
UFMG’s Pampulha campus map with units and gates showing the areas of questionnaire application.

The questionnaires were administered to 115 employees or users of the Pampulha campus, from 42 units/departments and five gates (Figure 4).

The detailed description of the questionnaire responses is provided in supplementary material. Most respondents (39.13%, 45/115) were gatekeepers and male (55.65%, 64/115). The age group with the highest number of participants was 32 to 38 years, followed by the 53 to 59 age group, which together accounted for 40.87% (47/115) of interviews. The most frequent level of education was completed high school (43.48%, 50/115), and the lowest was incomplete higher education (5.22%, 6/115). A large portion of respondents reported observing cats (83.48%, 96/115), and dogs (61.74%, 71/115) without owners in the unit or area where they work, a result consistent with the species distribution found in the campus resident population survey.

Regarding population management on campus, most respondents (70.43%, 81/115) believed that the university was responsible for the animals present in the units, considered it correct for the institution to assume responsibilities regarding them, and agreed with the animals residing on campus.

There was an association between believing that university assumed responsibility for the animals and considering it correct for the institution to do so (*p* = 0.016). Furthermore, there was an association between considering it correct for the university to assume responsibilities for the animals and agreeing with their presence on campus (*p* = 0.028). These associations demonstrate that most of the university community cares about the animals and agrees with institutional actions to promote animal welfare, characteristics of the work of CPPA-UFMG.

Regarding strategies to control the population of dogs and cats on university grounds, 55.65% (64/115) of respondents were in favor of removing the animals from campus, although this number was lower than the 92.17% who agreed with sterilization. Among those who disagreed with sterilization, reasons listed included not allowing the animal to have freedom or choice, as well as religious beliefs. Regarding the destination of the animals when removed from campus, many suggested handing them over to the city hall, non-governmental organizations (NGOs), or sending them for adoption. There was an association between favoring the remotion of animals from campus and being male (*p* = 0.034) with an age between 53 and 59 years (*p* = 0.006), as well as disagreeing with the method and being female (*p* = 0.034) with an age between 39 and 52 years (*p* = 0.006).

Concerning about ear tipping of cats and the use of collars with identification tags on dogs to recognize animals cared for on campus, 88.70% (102/115) of respondents agreed with the first method, and 95.95% (110/115) with the second, respectively.

A small proportion of respondents (8.7%, 10/115) had witnessed acts of animal cruelty in the unit or area where they worked, with the majority being commission against cats. Acts reported included threats, violence, and abandonment.

Regarding TNR most respondents (95.65%, 110/115) agreed that it is an adequate approach to reduce or prevent increasing in the population of dogs and cats on campus.

In relation to the return of animals to their original unit or area after surgical sterilization, 80% (92/115) of respondents agreed. There was a statistical association between agreeing with the return and the age of the respondent (*p* = 0.016), with an association between agreement and ages between 32 and 45 years and disagreement and ages between 46 and 52 years.

Finally, considering the concept of responsible pet ownership, more than 75% (88/115) were not familiar with the term, but when asked about knowledge related to crimes due to abandoning and mistreating animals, almost all respondents reported awareness.

## 4 Discussion

By 2018, actions related to abandoned animals on campus were carried out independently by professors and staff members who were concerned about the presence of sick and abandoned animals, in partnership with the Municipal Center for Zoonosis Control, using the T.N.R. methodology, supported by current state legislation. After reproductive and sanitary management, animals were put up for adoption, and when adoption did not occur, they were returned to the location where they were captures or rescued. However, the actions were not continuous, and reports of abandonment and mistreatment were constant.

From the creation of the Commission, actions were systematized in an integrated approach between the University and the municipal animal control service, ensuring constant surveillance, health, and welfare for the animals and the campus community.

A higher number of cats compared to dogs was observed at UFMG. The proportion of cats approached the studies conducted at the campuses of the University of Central Florida (25). The sex ratio of dogs differed from the ratio found in the study by Garcia et al. (26), in São Paulo, with animals in street situations, where the number of males was higher than that of females.

The proportion of adult animals on campus may indicate that the population is stable and without new births, as in other long-term T.N.R. programs, where the number of kittens in the colony was reduced to zero within a period ranging from 2 to 8 years (25).

All animals residing on campus were surgically sterilized. In units where all animals were sterilized, there were no puppies, in line with the information that the higher the sterilization rate, the lower the number of puppies (14).

According to Amaku et al. (27), for dogs, in management programs where high sterilization rates are applied, such as 80% per year, it would take a period of 5 years to decrease animal density, and according to Miller et al. (28) for cats, sterilizing 40% of the animals per year, in the long term would result in an accumulated sterilization rate of 75%. This could be achieved at UFMG if there were no high annual abandonment rates or measures that prevented the migration of animals from the regions near the campus. Therefore, education and surveillance actions must remain continuous and, if possible, be intensified each year.

In working done by Little et al. (29), testing animals for FIV and FELV in TNR programs is not recommended, since one of the premises of this type of population management involves sterilization, which prevents the transmission of FELV from the mother to the offspring and of both diseases in fights between males, important routes for maintaining the disease. Thus, the resource can be directed to the greatest possible number of sterilizations (29). In line with the recommendation, since December 2023, there has been no more testing of the cats managed by the UFMG commission, except for those that are referred for adoption.

Regarding the perception of campus employees, the associations found between believing that UFMG took responsibility for the animals, agreeing with their presence on campus, and considering it correct for the institution to manage the population of these species showed that most of the university community cares about animals and agrees with institutional actions to promote animal welfare, characteristics of CPPA-UFMG’s plan of action. Izaguirre and Montiel (30), in their perception study regarding animals on the campuses of the Autonomous University of Yucatan, Mexico, found that about half of the respondents believed that university authorities should be responsible for the dogs and cats residing on campuses.

Most of the employees were also in favor of removing animals from campus and delivering them to public or philanthropic shelters, with the majority being male, regarding the perception of strategies to control the population of dogs and cats on university grounds. The result is similar to the study conducted by Ash and Adams (31) at Texas A & M University, United States, and Kim et al. (32) in South Korea, where most respondents who agreed with animal removal as a management method were men. There was an association between disagreeing with animal removal and being a woman aged 39 to 52, which corroborates with the study conducted in Belgium, associating being female with a positive response to a community cat program on a university campus (33).

Regarding the forms of animal identification on campus, despite the recognition of collars in dogs achieving higher agreement, a significant fraction of respondents agreed with ear-tipping in cats, demonstrating an understanding and importance of this strategy. This practice, although widely adopted worldwide, faces resistance due to its aesthetic implications or consideration as an act of mutilation (34), which should not occur with the adequate anesthetic technique and local pain management.

There was greater agreement among respondents that T.N.R. would be a great strategy to decrease or prevent the increase in the population of dogs and cats on campus. There was no association with the gender of respondents among those who agreed with the methodology, unlike the results of other studies where more women supported T.N.R. as a method for population management (31, 35).

Few respondents were familiar with the concept of responsible ownership, but most knew that abandoning and mistreating animals are crimes. These data are important from the perspective of perception and association with injuries and mistreatment, as knowledge about responsible ownership is a factor that influences the reduction of domestic animal exposure to diseases (36), as well as in the expectation of effort for the management of an animal (37). Therefore, it is important to maintain education strategies focusing on responsible animal ownership (38).

## 5 Conclusion

The ethical population management of dogs and cats is a challenge for public health politics nowadays and should be based on evidence and indicators, so that the results can be measured and the evaluations of the efforts provide a basis for the next steps to be taken, since the management program must be a continuous, uninterrupted and transdisciplinary work.

Since its creation in 2018, CPPA-UFMG has been conducting actions for the ethical population management of dogs and cats on the university campuses. At the Pampulha campus, until the halfway point of implementation (September 2021), many animals, mostly cats, were recorded in 25 different units or areas. The effectiveness of surgical sterilization practices for approximately half of these animals observed during the period, and the nearly one-fifth of the animals being adopted, qualitative evaluation has become an essential tool for progress and the possibility of altering the implemented practices.

Perception research has shown that, despite the smaller portion of respondents being aware of the CPPA in the first year of implementation, the vast majority agreed with the T.N.R. methodology defined for the ethical population management of dogs and cats present on campus. Understanding what the human population perceives, which coexists with the animals in the spaces targeted by population management interventions, was important for directing educational actions to the most sensitive topics and ensuring success, reducing conflicts, and establishing collective agreements for the Program’s objectives.

After 5 years of implementation of the Management Program, it was observed, when compared to other studies, promising results that the actions at the Pampulha campus are successful, with the sterilization of 100% of residents and population stability.

In light of the results of this work, it is noted that people’s perception can align with what theory advocates, simply by giving a name to what is already present in the collective imagination, and when necessary, through education, planting the seed of cultural change that is expected, so that we can approach the world we envision, for ourselves, for the animals, and for the environment.

The challenges of ethical population management at the Pampulha campus of UFMG increase because, as the number of people involved in the work grows, so does surveillance and monitoring, which generate more occurrences to be addressed. Diversity in opinions and ways of acting also grows, inspiring better management of the human resources involved. Science is what should underpin the actions, but without humanity, there is no task worthy of effort. Listening to people and putting ourselves in their shoes leads us to see, in their way, the world that we are accustomed to seeing in our own way, and therefore, as Saramago taught, “to know things, you have to turn them all around.”

## Data availability statement

The original contributions presented in the study are included in the article/supplementary material, further inquiries can be directed to the corresponding author.

## Ethics statement

The studies involving humans were approved by Research Ethics Committee from UFMG by the protocol 3,356,456. The studies were conducted in accordance with the local legislation and institutional requirements. The participants provided their written informed consent to participate in this study. The animal studies were approved by Committee on Ethics in Animal Use (CEUA) from the UFMG by the protocol number 60/2022. The studies were conducted in accordance with the local legislation and institutional requirements. Written informed consent was obtained from the owners for the participation of their animals in this study.

## Author contributions

GB: Conceptualization, Formal analysis, Investigation, Methodology, Validation, Writing – original draft, Writing – review & editing. LO: Formal analysis, Validation, Writing – review & editing. CO: Conceptualization, Formal analysis, Investigation, Methodology, Supervision, Writing – original draft, Writing – review & editing. AC: Conceptualization, Validation, Writing – original draft. CB: Conceptualization, Writing – original draft, Validation. CT: Data curation, Methodology, Writing – review & editing. CM: Conceptualization, Writing – original draft. FS: Conceptualization, Writing – original draft. GL: Conceptualization, Writing – review & editing. LM: Data curation, Methodology, Writing – review & editing. LV: Writing – original draft. MC: Writing – original draft. RF: Writing – review & editing. VG: Writing – review & editing. WB: Data curation, Methodology, Writing – review & editing. YO: Data curation, Methodology, Writing – review & editing. DS: Conceptualization, Formal analysis, Investigation, Methodology, Supervision, Writing – original draft, Writing – review & editing.

## Footnotes

^1^ https://epitools.ausvet.com.au/oneproportion
